# Empiricism and Theorizing in Epidemiology and Social Network Analysis

**DOI:** 10.1155/2011/157194

**Published:** 2010-11-25

**Authors:** Richard Rothenberg, Elizabeth Costenbader

**Affiliations:** ^1^Institute of Public Health, Georgia State University, 140 Decatur Street, Atlanta, GA 30303, USA; ^2^Family Health International, Behavioral and Social Sciences Department, 2224 E NC Hwy 54, Durham, NC 27713, USA

## Abstract

The connection between theory and data is an iterative one. In principle, each is informed by the other: data provide the basis for theory that in turn generates the need for new information. This circularity is reflected in the notion of abduction, a concept that focuses on the space between induction (generating theory from data) and deduction (testing theory with data). Einstein, in the 1920s, placed scientific creativity in that space. In the field of social network analysis, some remarkable theory has been developed, accompanied by sophisticated tools to develop, extend, and test the theory. At the same time, important empirical data have been generated that provide insight into transmission dynamics. Unfortunately, the connection between them is often tenuous and the iterative loop is frayed. This circumstance may arise both from data deficiencies and from the ease with which data can be created by simulation. But for whatever reason, theory and empirical data often occupy different orbits. Fortunately, the relationship, while frayed, is not broken, to which several recent analyses merging theory and extant data will attest. Their further rapprochement in the field of social network analysis could provide the field with a more creative approach to experimentation and inference.

## 1. Introduction

Theory and empirical data are in principle intimately interwoven. Yet in the practice of social network analysis, there appears to be a disconnect: theorizing and empiricism often seem to occupy separate orbits, and these separate discussions may be difficult to relate to each other. The root of the problem may lie in the different skill sets required by each, or perhaps in the substantial obstacles to collection of human network data. The following exploration of the distance between theory and empiricism suggests that a rapprochement would be of considerable benefit to the field.

The mid-19th Century American philosopher Charles Peirce coined the term “abduction” (which he also called “retroduction”) to fill a gap he perceived in the territory occupied by induction and deduction. As distilled by Professor Burch [1], Peirce used syllogisms to explain this term, substituting Rule, Case, and Result for the more familiar Major Premise, Minor Premise, and Conclusion. But perhaps more interesting to epidemiologists and social network analysts, he related this logical process to sampling. As Professor Burch explains it, a standard valid syllogism would progress as follows.

*Rule*: All balls in this urn are red.
*Case*: All balls in this particular random sample are taken from this urn.
*Result*: Therefore, all balls in this particular random sample are red.

Peirce then asked what would happen if we change the order of reasoning, by interchanging the Result and the Rule.

*Result*: All balls in this particular random sample are red.
*Case*: All balls in this particular random sample are taken from this urn.
*Rule*: Therefore, all balls in this urn are red.

Burch points out that this is not a valid syllogism but was the core of Peirce's concept of induction. Extraordinary, how closely it captures the epidemiologic mindset. But take it one step farther, and interchange the Result with the Case.

*Rule*: All balls in this urn are red.
*Result*: All balls in this particular random sample are red.
*Case*: Therefore, all balls in this particular random sample are taken from this urn.

Again, not a valid construct, but if we substitute “Alternate Hypothesis” for “Rule,” we appear to capture the essence of hypothesis testing as it is now practiced [2]. Burch maintains that this is neither induction nor deduction, but a new type of argument that Peirce called abduction. Peirce went on to use the three “-ductions” to describe the scientific method as a *circular synthesis* of the scientific method. The process begins with a conjecture or hypothesis that is based on some observation or thought (abduction). From the hypothesis can be derived consequences, and these can be tested. The resulting test observations can be used to confirm or refute the hypothesis, or more generally, either to draw conclusions about the truth or return to the abductive process of conjuring up a new hypothesis.

Popper did not agree [3]. He relegated the process of hypothesis generation to the realm of psychology and stated overtly that he was not interested in it [3, page 39]. In contrast, Albert Einstein embraced it. As described by Adam [4], Einstein wrote a short newspaper article in 1919 that colocated the process of abduction with the creativity inherent in scientific endeavors. Einstein said: “Intuitive comprehension of the essentials about the large complex facts leads the researcher to construct one or several hypothetical fundamental laws…he [the researcher] does not arrive at his system of thought in a methodical, inductive way; rather, he snuggles (sic) to the facts by intuitive choice among the imaginable axiomatic theories.”

Thus, Peirce and Einstein provide a direct connection between theory, observations, conclusions, and revisions. This view stresses that theory and observation are interdependent, iterative, and connected by creativity. Unfortunately, this connection (though not necessarily the creativity) seems to have attenuated in the application of social network analysis to disease transmission.

## 2. The Linkage of Theory and Empiricism

Several factors have hindered a tight linkage between theoretical and empirical approaches. First, the cost and time to elucidate sociometric network structure, particularly for hard-to-reach populations such as those who may be at the highest risk for HIV or other communicable diseases, are often viewed as prohibitive. Second, empirical sociometric network ascertainment is imperfect. Since the boundaries of the populations of interest are never known and always changing and the manner in which we find out about connections is not standardized, some connections between individuals or network nodes within those populations are always missed, often in unknown ways that render imputation and interpretation problematic. Third, there is no gold standard and no true or known network against which to measure empirical adequacy. These concerns are all subsumed under the general issue of sampling in networks. Because empirical ascertainment of networks requires a credible sampling procedure, preferably one that justifies the use of standard statistical theory, observations may be suspected. One result has been a movement toward theory-based network simulation wherein the investigator controls the sampling, knows (actually creates) the gold standard, and can test the effect of imposed conditions. The past decade has witnessed a burgeoning of this work and considerable new insight into the structure, function, and dynamics of many types of networks [5, 6]. 

A persistent problem, however, is the difficulty of relating theoretical network constructs back to some empirical reality. The theoretical biases inherent in sampling are the case in point. There can be no question that sampling matters if one is to have a credible mathematical basis for statistical network inference [7, 8]. Modeling approaches have demonstrated the biases that arise from missing data [9]. In his text, Newman [10] enumerates some of these biases: snowball sampling finds persons in proportion to their eigenvector centrality (i.e., the centrality of their contacts), but the large number of waves required to reach equilibrium may preclude unbiased estimates. Contact tracing suffers from the same problem, with the additional issue of seeking only infected persons, who are a biased sample of the population. Random walk sampling may offer some advantages, since sampling is proportional to degree, and equilibrium can be reached quickly in small groups, but issues of contact recall, unfindable partners, and nonparticipation persist. These assertions are all readily verifiable using mathematical and simulation approaches. There has been little or no empirical validation, however, of many theoretical conclusions that are taken as true. In fact, the assumption of theoretical validity is often so strong that many may find empirical verification unnecessary.

## 3. Reconnecting Theory to Data

 But if the Peirce/Einstein view is to be recaptured, meaningful efforts at falsification of theoretical constructs are needed. As noted, such efforts are generally not attempted, perhaps because of their difficulty, or perhaps because of the *a priori* assumptions about their inadequacy. (You cannot know if you have the right answer, so why bother.) This is perhaps where Peirce's second syllogism—the balls in my random sample are all red, so those in the urn from which they come must be red—needs to be invoked. Though logically defective—in fact, it epitomizes “the inductive problem” that has concerned philosophers since Hume—it is the basis for the inductive reasoning that, as noted, drives the epidemiological mindset. As argued forcefully by Pearce and Crawford-Brown [11], the notion that falsifiability is the hallmark of science fails to recognize the uncertainties of falsifiability, which can be at least as strong as those of induction. In addition, these authors stress the primacy of replication and validation of findings [12], the need for mature theory examined in multiple ways, and the importance of observations whose ongoing renewal and explanation is actually the work of theory. 

Thus, to complete the loop of theory validation, we require repeated demonstration that theoretical predictions are borne out in real life. Empirical verification of theoretical constructs affirms their validity, provides ongoing refinement of parameters, and furnishes a real basis for applying interventions. In the current realm of social network analysis, it would seem that empirical studies provide parameters to theoreticians, and not much else.

## 4. Some Other Examples

On the other hand, it is also the case that those involved in delineating real-time social networks have focused more on findings and transmission implications than on the specific validation of theoretical constructs. For example, 15 empirical network studies that were used in a synthesis of findings [13] produced over 100 publications, but none focused primarily on testing theoretical findings. There are some examples, however, of empirical attempts to examine theoretical constructs. Take, for example, Newman's assertion that, with random walk sampling, equilibrium can be reached quickly in small groups. Two empirical observations speak to this issue. First, in a direct test of sampling methods [14], networks ascertained by a chain link random walk (wherein the next person in the chain was chosen at random from the contacts of the current respondent) or by nomination (the next person in the chain nominated by the respondent from his/her contacts) were indistinguishable. Second, using those same networks, the underlying pattern of network configuration was evident from the first 10 interviews (out of 206) (Table 1), supporting the notion that the pattern becomes clear quickly. 

In a comparison of centrality measures [15], it was demonstrated that imperfect sample data produced stable network estimates under a variety of circumstances. In a comparison of eight types of centrality measures, high concordance [16] was found among measures ascertained through a complex, mixed sampling scheme despite expectations that these measures would vary because of their differing relationships to the underlying sampling method. 

A number of studies, following the observations by Barabási and colleagues of “scale-free” network structure in the world wide web [17–19], attempted to show that networks of persons at risk for HIV and STIs could be fit by a power law curve with a coefficient between 2 and 3 (the statistical requirement for scale-freeness) [13, 20]. Several rigorous statistical analyses [7, 21] of the empirical data from 10 studies found that none of the nine statistical models tested consistently provided the best fit to the degree distributions from those studies. In addition, the best-fit power law model predicted no epidemic threshold for HIV and STIs in the United States, a theoretical observation in obvious contrast to the true condition. This result [21], by providing empirical evidence against the proposed theory, embodies the aforementioned process of “circular synthesis.”

As a final example, the history of concurrency as an important feature of HIV and STD transmission is informative. Though disjointed, and at times acerbic, the discussion has gone back and forth between theory and data and provides a good illustration of how the two interact. The role of concurrency in Africa was first suggested nearly 20 years ago, based both on observation [22, 23] and on theoretical considerations and simulation [24]. In a comprehensive followup [25–27], mathematical development of a simple formula for calculating network concurrency and a simple simulation established the importance of concurrency in transmission. Ten years on, extensive claims have been made for the overriding importance of concurrency in sexual transmission of HIV in Africa [28, 29], with the assertion that multiple sites, assessed in multiple ways, have evidence of substantial concurrency. Though the empirical evidence for these claims has been challenged [30, 31], and the challenge contested [32], the pattern of high long-term concurrency with a relatively low degree distribution has been demonstrated in detail in at least one comprehensive study, in Likoma Island, Malawi [33]. This nonlinear chain of events does nonetheless illustrate the importance of the interplay between conjecture, empirical data, and theoretical development. The next step, not yet completed, would be a theoretical demonstration of rapid epidemic spread in an African setting that would incorporate a low-degree high concurrency configuration and reasonable parameters for transmission based on emerging empirical information on infectivity in acute HIV infection [34]. (In another aspect of concurrency—its potential role in explaining the ethnic disparity in HIV infection in the United States—this type of theoretical and empirical interplay has been attempted to confirm its importance [35].)

## 5. Interlocking Roles

Though there are other examples of the circular process of empirical and theoretical interaction, they are still few in number. The majority of empirical studies (e.g., large-scale surveys) from which parameters are drawn are usually theory-free. In turn, theoretical and simulation studies, as noted, use these parameters but are often data- and context-free. (An unfair characterization, perhaps, but it is difficult to deny that ethnographers generally do not speak mathematics and mathematicians do not speak the language of the street.)

But from these considerations, a clearer role for theory, empiricism, and their interrelationship may emerge. In his Nobel acceptance speech in 1974, Frederich von Hayek, often called the father of complexity theory, said: “…*as we penetrate from the realm in which relatively simple laws prevail [the physical sciences] into the range of phenomena where organized complexity rules…often all that we shall be able to predict will be some abstract characteristic of the pattern that will appear…yet…we will still achieve predictions which can be falsified and which therefore are of empirical significance*” [36]. Despite all their difficulties, empirical descriptions of networks, both qualitative and quantitative, have the potential to find those abstract characteristics of a pattern, a task for which theoretical and simulation studies alone are not well suited. Theoretical studies are well suited to exploring patterns, and they often do it best in ways that make little pretense of reality [37] but are geared rather to demonstrating mechanisms and testing the observations. A greater synergy between theory and data could provide the field with a more systematic approach to experimentation and inference.

Fortunately the process of abduction is a method equally approachable by all scientists. Theoreticians can be just as good abductors as empiricists. Anyone is at liberty to think up ideas, but those who “snuggle to the facts” may have the best chance of success.

## Figures and Tables

**Table 1:** Network features with chronological accrual of respondents.

Number of respondents	10	20	30	40	50	100	150	All (206)
Number of persons in network	62	131	202	284	367	685	981	1314
Degree (mean of interviewed respondents)	7.4	8.1	8.3	8.6	8.8	8.4	8.0	7.6
Degree (mean of all persons in network)	2.3	2.3	2.3	2.3	2.3	2.3	2.3	2.3
Degree (variance)	10.5	13.4	13.1	12.8	12.5	12.1	11.8	10.9
Concurrency (kappa)	5.9	7.1	7.0	6.9	6.8	6.6	6.4	6.1
Clustering coefficient	0.034	0.034	0.033	0.029	0.028	0.033	0.041	0.036
Power coefficient	2.79	2.19	2.23	1.76	1.71	1.65	1.59	1.59
Age assortativity	0.313	0.299	0.348	0.315	0.285	0.329	0.323	0.319
